# Accuracy of Commercial Molecular Diagnostics for the Detection of Pulmonary Tuberculosis in China: A Systematic Review

**DOI:** 10.1038/s41598-019-41074-8

**Published:** 2019-03-14

**Authors:** Siwei Deng, Yixin Sun, Hui Xia, Zhike Liu, Le Gao, Jichun Yang, Yanlin Zhao, Fei Huang, Jingnan Feng, Lixia Wang, Shitong Huan, Siyan Zhan

**Affiliations:** 1 Department of Epidemiology and Biostatistics, Peking University School of Public Health, Beijing, China; 2 National Center for Tuberculosis Control and Prevention, Chinese Center for Disease Control and Prevention, Beijing, China; 3 Bill and Melinda Gates Foundation, Beijing, China

## Abstract

This systematic review assesses the accuracy of molecular diagnostic methods for the detection of pulmonary tuberculosis in studies performed in China, published in Chinese and English. We searched for studies that assessed the accuracy of molecular diagnostics for pulmonary TB in China in the China National Knowledge Infrastructure, the Wanfang Database, SinoMed, VIP Information, Pubmed, Embase, and the Cochrane Library. For each index test, a summary estimation for sensitivity and specificity was calculated using the bivariate random-effects model. A total of 59 studies were included in our analysis. Loop-mediated isothermal amplifcation (LAMP) assay (six studies; pooled sensitivity 90%, 95% CI 78–95%; specificity 93%, 85–97%), line probe assay (LPA) (one study; 87%, 84–90%; 94%, 92–95%) and polymerase chain reaction (PCR) (FQ-PCR and RT-PCR) (four studies; 90%, 55–99%; 93%, 71–99%) showed good diagnostic performance in the meta-analysis. The highest pooled sensitivity was from Xpert MTB/RIF (20 studies; pooled sensitivity 91%, 95% CI 87–94%). The highest pooled specificity was from cross-priming amplification (CPA) (six studies; pooled specificity 97%, 95–99%). The lowest pooled sensitivity and specificity were from simultaneous amplification and testing (SAT)-TB (three studies; 79%, 66–88%; 72%, 48–88%). In subgroup analysis, molecular diagnostics demonstrated higher sensitivity for pulmonary TB detection in smear-positive specimens. Xpert MTB/RIF, LAMP, LPA, CPA and PCR demonstrated high accuracy overall for pulmonary tuberculosis detection, while SAT-TB had poor performance.

## Introduction

Tuberculosis (TB) is a global public health concern. There were an estimated 10.4 million new cases of TB, and it caused 1.7 million deaths in 2016^1^. China has a high TB burden, with 64 new cases and 3.6 deaths per 100,000 population in 2016^1^. TB is curable if it is treated in a timely manner, so the accurate and rapid detection of TB has a critical role to play in the prevention and treatment of TB^2^.

Currently, there are both direct and indirect methods of diagnosis of TB. Direct detection methods include microscopy, culture, antigen detection, and nucleic acid detection; indirect tests include immune response assessment with the tuberculin skin test (TST) and interferon–gamma release assays (IGRAs). Sputum smear microscopy is cheap and easy to operate, but its sensitivity and specificity are not good^3^. Mycobacterial culture may be the most reliable reference standard, but it has the disadvantage of low efficiency and usually takes several days for the results to be produced^4^. The sensitivity of TST and IGRAs is suboptimal, and neither adequately distinguish latent from active TB^5^. Molecular diagnosis, a new direct method, has the advantages of simplicity, rapidity, and accuracy. It is urgent and imperative for this method to be promoted in China. The CFDA has endorsed 10 types of molecular diagnoses of TB, including Xpert MTB/RIF, loop-mediated isothermal amplifcation (LAMP) assay, line probe assay (LPA), Genechip (biochip), MeltPro TB assay, RealAmp, cross-priming amplification (CPA), simultaneous amplification and testing (SAT)-TB, Genprobe AMTD, and polymerase chain reaction (PCR) (FQ-PCR and RT-PCR).

Several validation studies of molecular diagnoses for TB have been published in Chinese^6–9^. To inform the policy decisions of the Chinese CDC and the Ministry of Health, it is necessary to summarize the accuracy of those studies. This systematic review assesses the diagnostic accuracy of molecular diagnosis for pulmonary TB in China.

## Results

### Search results

**Figure 1:**
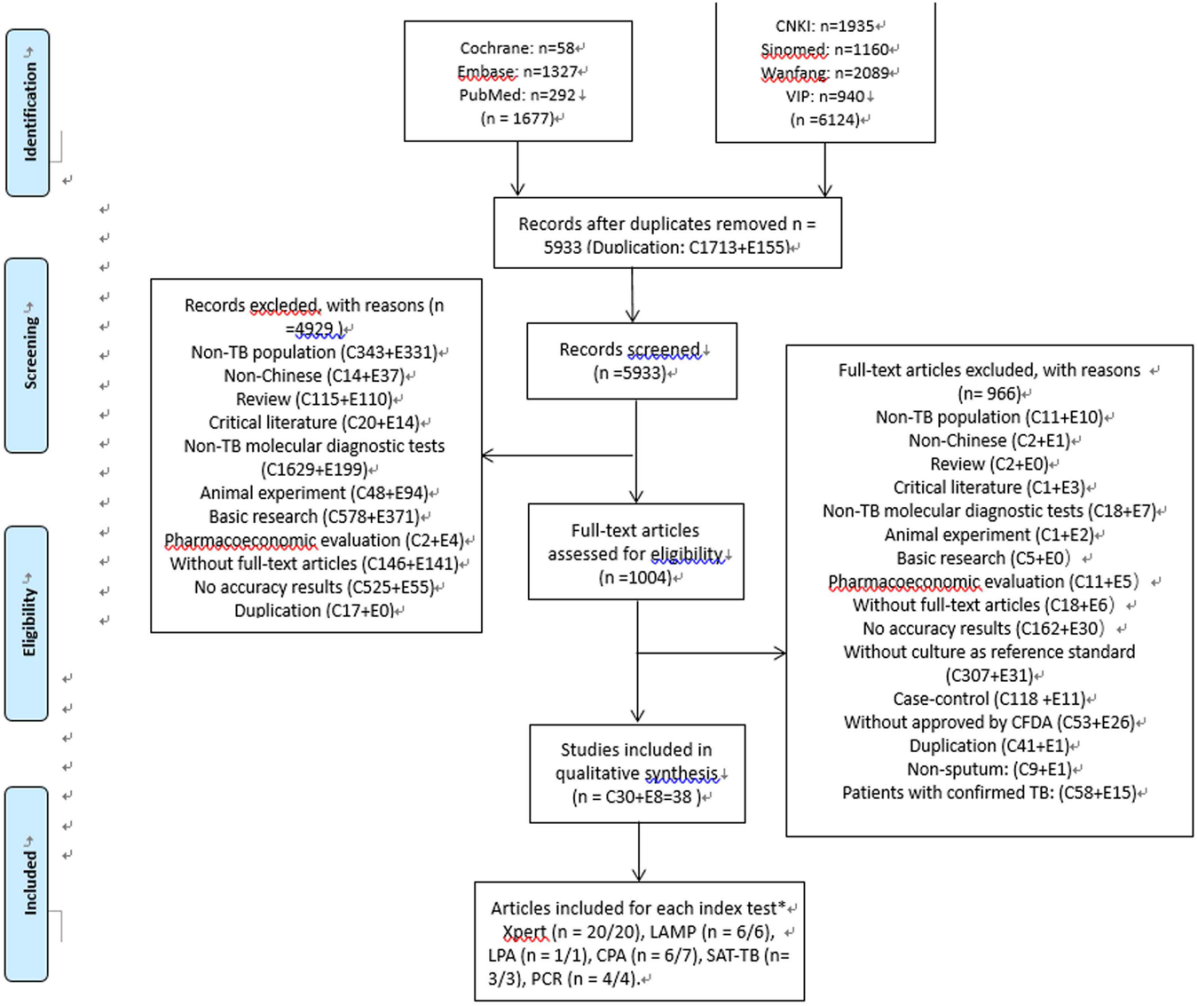
Study flow diagram of studies in the review.

Our search identified 7801 papers. After being filtered in the study selection process (Fig. 1), 38 articles were included in the final analysis. Among these, two articles reported more than one surveys done using different index tests but with the same procedures; these data were extracted separately and counted as distinct studies. Appendix 3 exhibits the characteristics of the 41 studies. All studies were independent validation studies, with 33 published in Chinese and the others published in English. There were 23 cohort studies and 18 cross-sectional studies, and 28 studies were performed at city- and province-level laboratories. Solid media and liquid media were used in 25 and 11 studies respectively, with no report in 5 studies. Only 16 studies indicated the population type: 8 with adults, 1 with children and 7 with both adults and children. None of these studies reported the HIV infection status of the subjects.

The CFDA had approved six of the commercial molecular diagnostic methods used in the studies examined in our meta-analysis, including Xpert MTB/RIF (n = 20 articles/20 studies), LAMP (n = 6 articles/6 studies), LPA (n = 1 articles/1 studies), CPA (n = 6 articles/7 studies), SAT-TB (n = 3 articles/3 studies) and PCR (n = 4 articles/4 studies). Sample sizes ranged from 47 to 3151.

### Methodological quality of included studies

**Figure 2:**
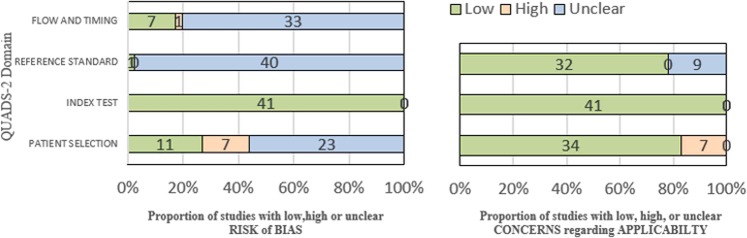
Quality Assessment of included articles.

The results of the quality assessment are shown in Fig. 2. Of the 41 studies, 11 were considered to have low risk patient selection, while 7 were judged to be high risk because they preselected smear-positive patients or other specific subgroups of suspected tuberculosis. The remaining 23 studies were judged to be unclear because the manner of patient selection was unclear. In the index test domain, all studies were considered low risk. All studies except one, which was considered low risk, were judged to have unclear risk in terms of reference standard because they did not report whether the people performing the reference tests were blinded to the results of the index tests. Regarding flow and timing, 33 studies were judged to have unclear risk because they did not state the time interval between the index test and reference standard. Regarding applicability, in patient selection, there were 34, 7, and 0 studies considered low risk, high risk and unclear, respectively. All studies were judged to be low risk in their index tests. In terms of the reference standard domain, 32 and 9 studies were considered low risk and unclear, respectively. The quality assessment details for each study are presented in Appendix 4.

**Figure 3:**
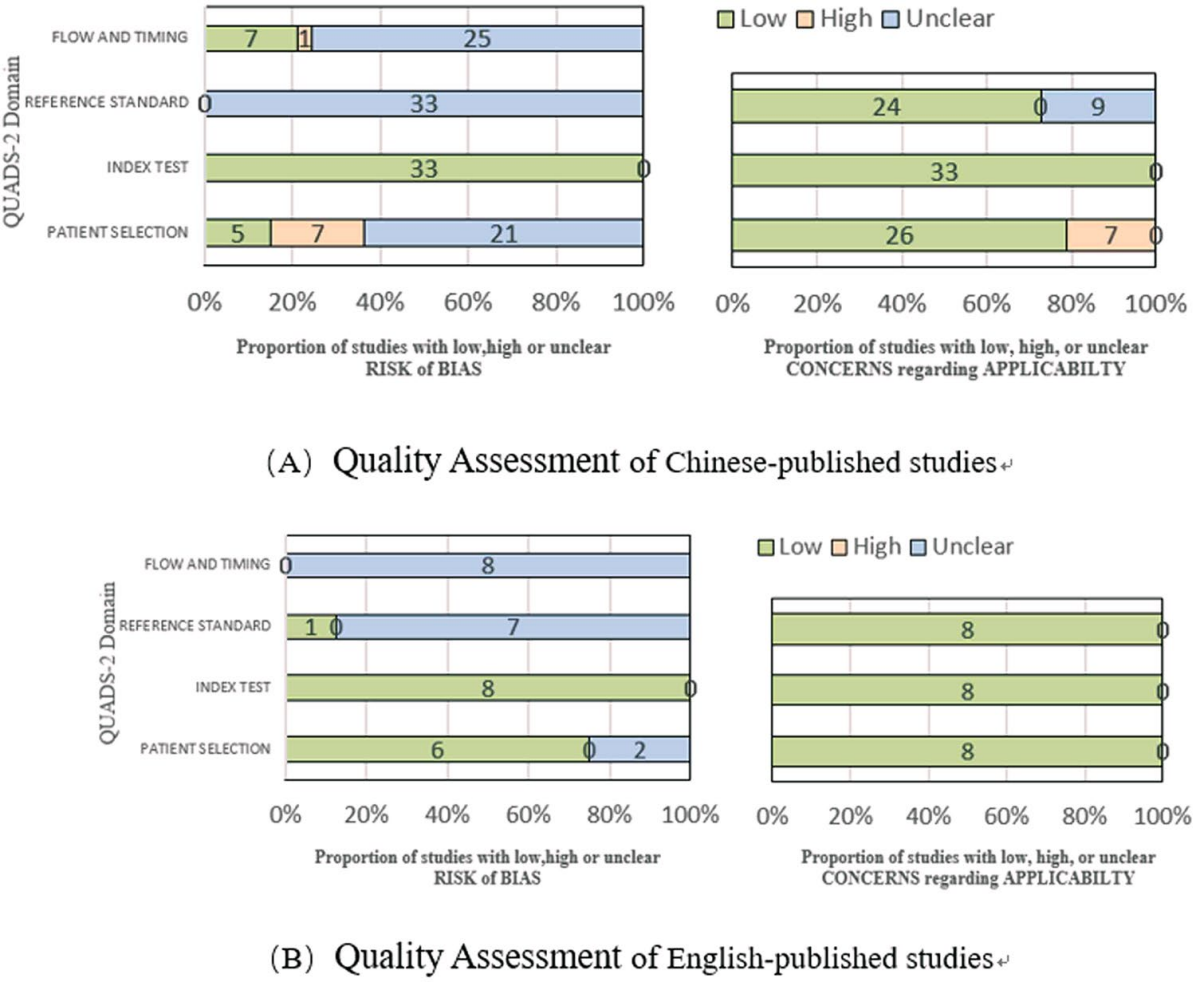
Quality Assessment Results of Chinese-published and English-published studies for TB.

Some differences were found between the quality of the studies published in Chinese and of those published in English, so we assessed the quality of these groups of studies separately. The results are showed in Fig. 3. The articles published in English showed a lower risk of bias, both in patient selection and reference standard, than the ones in Chinese.

### Diagnostic test accuracy

#### Xpert MTB/RIF

**Figure 4:**
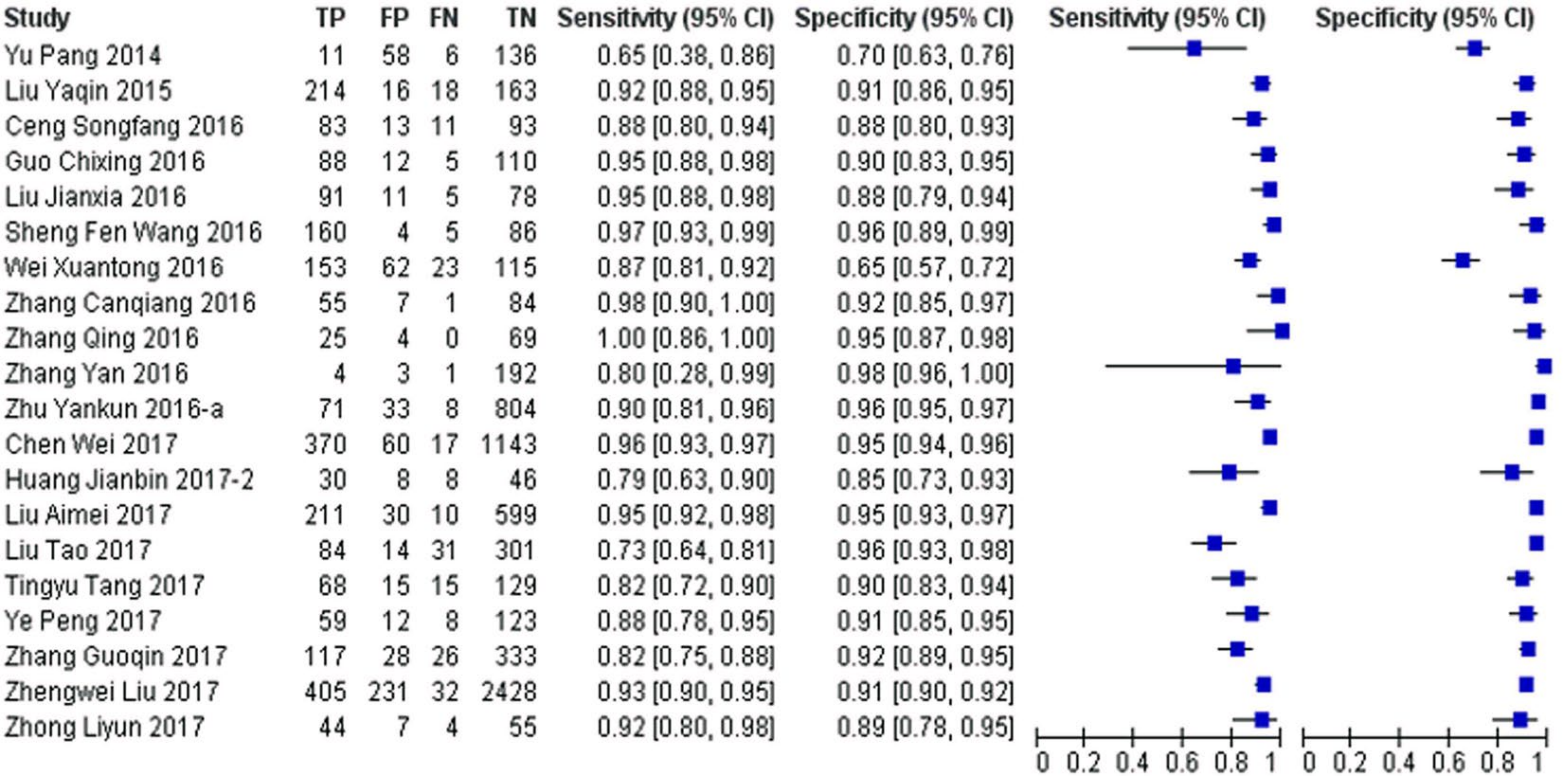
Forest plots of sensitivity and specificity for Xpert MTB/RIF assay. The pooled sensitivity was 91% (95% CI 87–94%; n = 20); pooled specificity of all studies was 92% (95% CI 89–94%; n = 20). TP = true positive; FP = false positive; FN = false negative; TN = true negative. The point estimates of sensitivity and specificity from each study are shown as solid circles (blue square). Error (black horizontal line) bars indicate 95% CI. Numbers indicate the studies included in the meta-analysis, as cited in the reference list.

**Figure 5:**
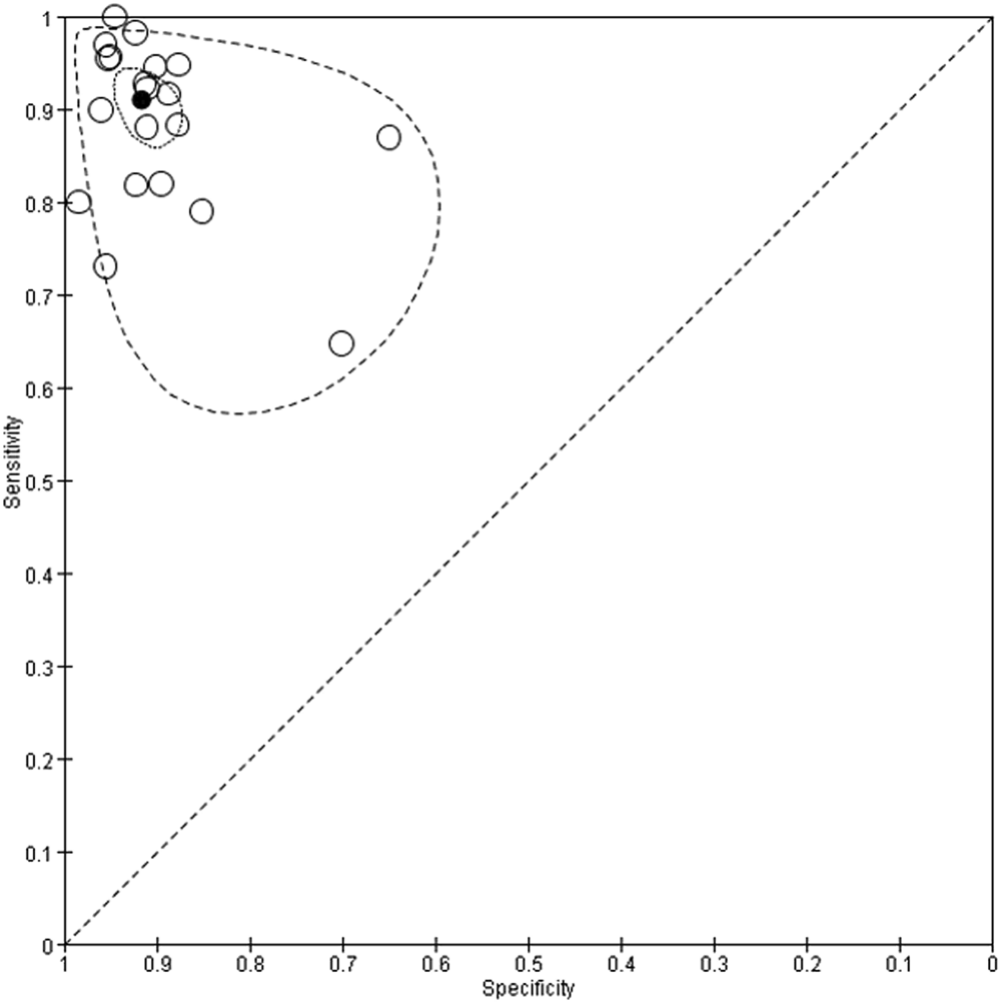
Summary receiver operating characteristic (SROC) curves for Xpert MTB/RIF assay. Each individual study is represented by an empty circle. The size of the circle is proportional to the sample size of the study. The solid circles correspond to the summary estimates of sensitivity and specificity and are shown with 95% confidence regions (dotted lines) and 95% prediction regions (dashed lines).

The diagnostic accuracy of Xpert MTB/RIF (Cepheid, United States) was reported in 20 studies (10392 participants). The pooled sensitivity was 91% (95% CI: 87–94%) and specificity was 92% (95% CI: 89–94%) (Figs 4, 5).

Two studies reported the accuracy of smear-positive TB. Because of the small sample size, we retained the figures for the sensitivity and specificity of the original studies. The sensitivities were 100% (95% CI: 91–100%) and 98% (95% CI: 94–99%) respectively (Table 1). The specificity of the smear-positive patients had no meaning, so we do not report it. Four studies reported the accuracy of smear-negative TB, and the pooled sensitivity was 75% (95% CI: 52–90%) and the specificity was 94% (95% CI: 81–98%). (Table 1). The sensitivity of the Xpert method was higher in smear-positive patients than in smear-negative patients.

**Table 1:** Subgroup analysis of molecular diagnostics results for diagnosing TB.

Molecular diagnostics	Sputum Smear	No. of studies	Pooled Sensitivity [95% CI]	Pooled Specificity [95% CI]
①Xpert MTB/RIF	Positive	2*^,a^	1.00 [0.91, 1.00] 0.98 [0.94, 0.99]	—
	Negative	4*^,b^	0.75 [0.52, 0.90]	0.94 [0.81, 0.98]
②LAMP	Positive	2*^,a^	0.92 [0.87, 0.96] 0.97 [0.93, 0.99]	
	Negative	3*^,c^	0.67 [0.49, 0.81]	0.96 [0.86, 0.99]
③LPA	Positive	0	—	—
	Negative	1*^,a^	0.87 [0.84, 0.90]	0.94 [0.92, 0.95]
④CPA	Positive	1*^,a^	0.94 [0.91, 0.96]	
	Negative	1*^,a^	0.82 [0.75, 0.88]	0.90 [0.87, 0.92]
⑤SAT-TB	Positive	1*^,a^	0.85 [0.81, 0.88]	
	Negative	1*^,a^	0.57 [0.52, 0.62]	0.59 [0.56, 0.62]
⑥PCR	Positive	1*^,a^	0.95 [0.87, 0.99]	
	Negative	1*^,a^	0.61 [0.36, 0.83]	1.00 [0.92, 1.00]

*^,a^The sensitivity and specificity of original studies were retained when the number of studies were less than 3.

*^,b^Bivariate random effect model to estimate the pooled sensitivity and specificity.

*^,c^Univariate random effect model to calculate the pooled sensitivity and specificity separately.

#### Loop-mediated isothermal amplification (LAMP)

**Figure 6:**
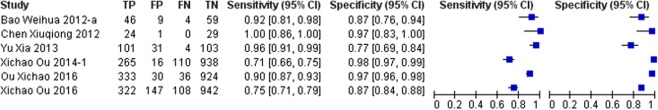
Forest plots of sensitivity and specificity for LAMP. The pooled sensitivity was 90% (95% CI 78–95%; n = 6); pooled specificity of all studies was 93% (95% CI 85–97%; n = 6). TP = true positive; FP = false positive; FN = false negative; TN = true negative. The point estimates of sensitivity and specificity from each study are shown as solid circles (blue square). Error (black horizontal line) bars indicate 95% CI. Numbers indicate the studies included in the meta-analysis, as cited in the reference list.

**Figure 7:**
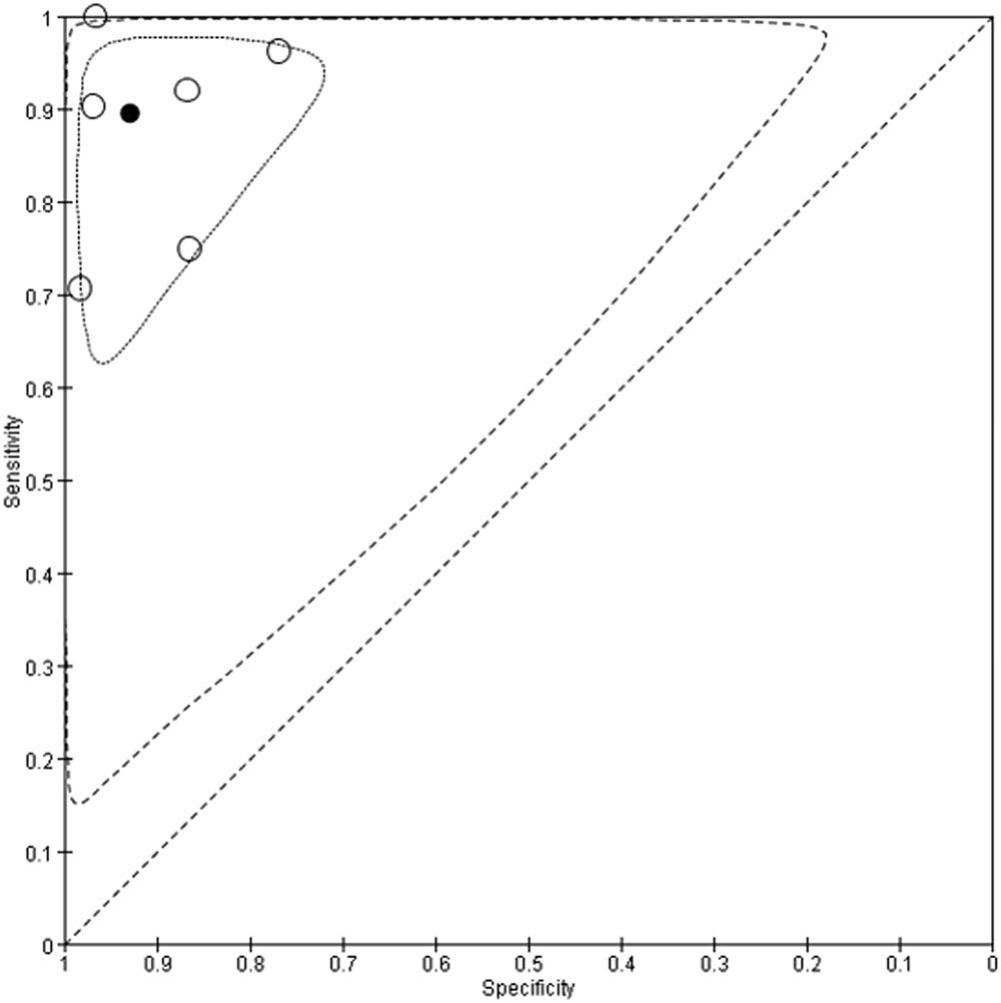
Summary receiver operating characteristic (SROC) curves for LAMP. Each individual study is represented by an empty circle. The size of the circle is proportional to the sample size of the study. The solid circles correspond to the summary estimates of sensitivity and specificity and are shown with 95% confidence regions (dotted lines) and 95% prediction regions (dashed lines).

A total of six studies (4653 participants) showed the diagnostic accuracy of LAMP. There were three manufacturers that produced the LAMP kits used: Eiken Chemical Company, Japan; Di’ao Biotech, China; and Hfbiotech, China. The pooled sensitivity and specificity (95% CI) were 90% (78–95%) and 93% (85–97%), respectively (Figs 6, 7).

Of the studies that performed subgroup analysis based on sputum smear status, two reported the sensitivity of smear-positive TB, which were 92% (95% CI: 87–96%) and 97% (95% CI: 93–99%) (Table 1). Three studies that evaluated the accuracy of smear-negative TB, reported a pooled sensitivity (95% CI) of 67% (49–81%) and a pooled specificity of 96% (86–99%) (Table 1).

#### Line probe assay (LPA)

**Figure 8:**
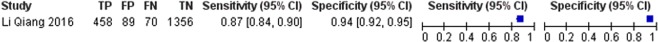
Forest plots of sensitivity and specificity for LPA. The sensitivity was 87% (95% CI 84–90%; n = 1); specificity of all studies was 94% (95% CI 92–95%; n = 1). TP = true positive; FP = false positive; FN = false negative; TN = true negative. The point estimates of sensitivity and specificity from each study are shown as solid circles (blue square). Error (black horizontal line) bars indicate 95% CI. Numbers indicate the studies included in the meta-analysis, as cited in the reference list.

Only one article (1973 participants) assessed the accuracy of LPA (Hain Lifescience, Germany). The sensitivity and specificity was found to be 87% (95% CI: 84–90%) and 94% (95% CI: 92–95%) (Table 1, Fig. 8).

#### Cross-Priming Amplification, EasyNAT®TB (CPA)

**Figure 9:**
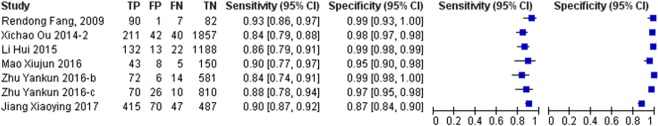
Forest plots of sensitivity and specificity for CPA. The pooled sensitivity was 87% (95% CI 84–89%; n = 7); pooled specificity of all studies was 97% (95% CI 95–99%; n = 7). TP = true positive; FP = false positive; FN = false negative; TN = true negative. The point estimates of sensitivity and specificity from each study are shown as solid circles (blue square). Error (black horizontal line) bars indicate 95% CI. Numbers indicate the studies included in the meta-analysis, as cited in the reference list. *^,a ‘^Zhu Yankun 2016-bc, the population of 2 studies were different.

**Figure 10:**
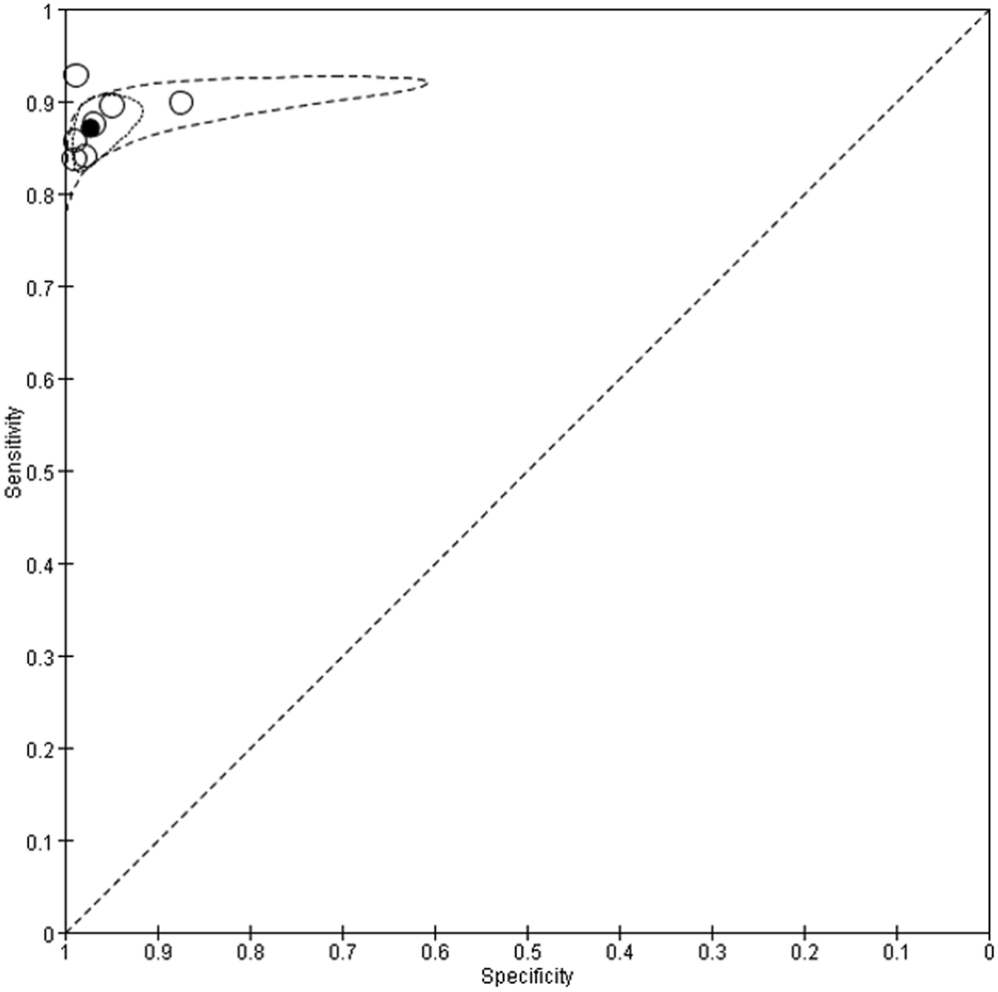
Summary receiver operating characteristic (SROC) curves for CPA. Each individual study is represented by an empty circle. The size of the circle is proportional to the sample size of the study. The solid circles correspond to the summary estimates of sensitivity and specificity and are shown with 95% confidence regions (dotted lines) and 95% prediction regions (dashed lines).

Seven studies (6551 participants) reported the results of CPA (Ustar Biotechnologies Co., Ltd, China) accuracy. Sensitivities ranged from 84% to 93%, with pooled estimates of 87% (95% CI: 84–89%). Specificities ranged from 87% to 99%, with pooled estimates of 97% (95% CI: 95–99%) (Figs 9, 10).

One study reported the status of sputum smear. For smear-positive TB, the sensitivity was 94% (95% CI: 91–96%) (Table 1), and for smear-negative TB, the sensitivity and specificity were 82% (95% CI: 75–88%) and 90% (95% CI: 87–92%), respectively (Table 1).

#### Simultaneous Amplification and Testing (SAT)-TB

**Figure 11:**
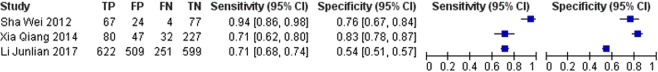
Forest plots of sensitivity and specificity for SAT-TB. The pooled sensitivity was 79% (95% CI 66–88%; n = 3); pooled specificity of all studies was 72% (95% CI 48–88%; n = 3). TP = true positive; FP = false positive; FN = false negative; TN = true negative. The point estimates of sensitivity and specificity from each study are shown as solid circles (blue square). Error (black horizontal line) bars indicate 95% CI. Numbers indicate the studies included in the meta-analysis, as cited in the reference list.

**Figure 12:**
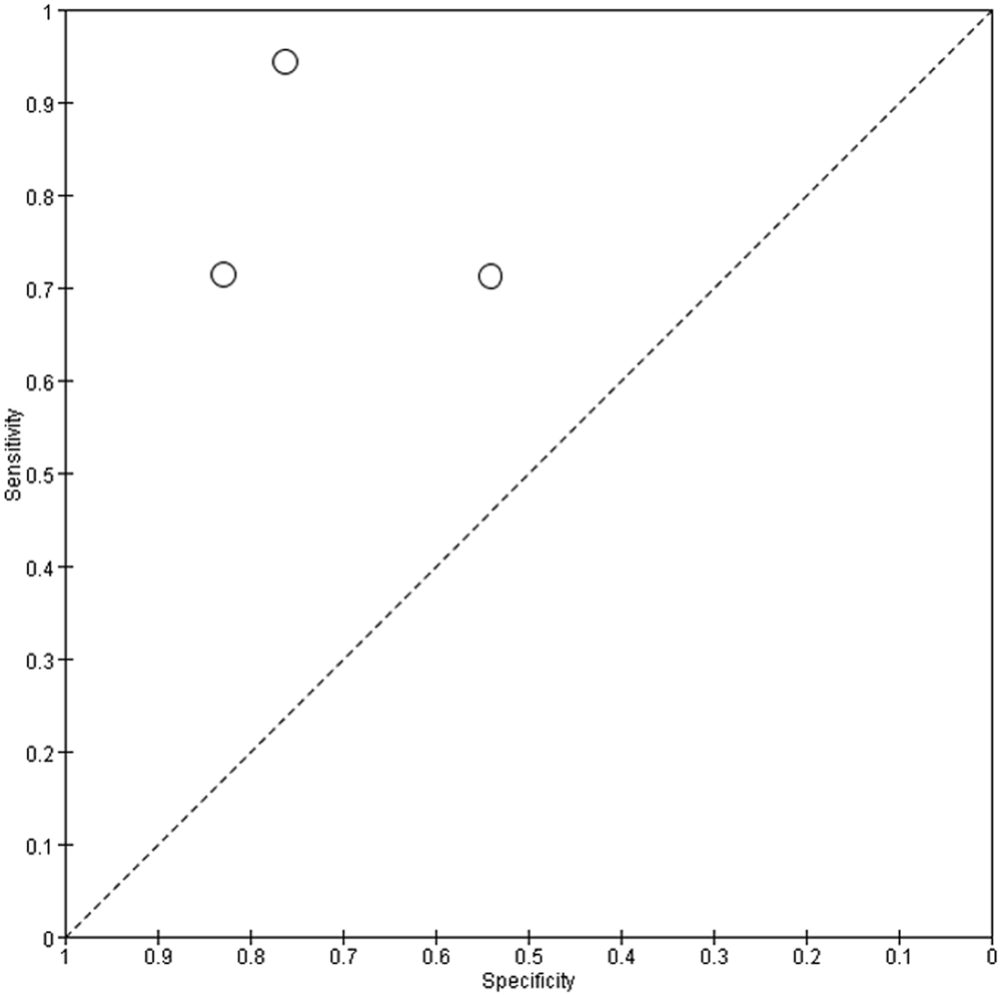
Summary receiver operating characteristic (SROC) curves for SAT-TB. Each individual study is represented by an empty circle. The size of the circle is proportional to the sample size of the study. The solid circles correspond to the summary estimates of sensitivity and specificity and are shown with 95% confidence regions (dotted lines) and 95% prediction regions (dashed lines).

Three studies (2939 participants) reported the sensitivity and specificity of SAT-TB (Rendu Biotechnology, China) in the detection of TB. The pooled sensitivity and specificity (95% CI) determined by univariate analyses were 79% (66–88%) and 72% (48–88%) (Figs 11, 12).

Only one study focused on sputum smear status. The sensitivity of smear-positive TB was 85% (95% CI: 81–88%) (Table 1), and the sensitivity and specificity of smear-negative TB were 57% (95% CI: 52–62%) and 59% (95% CI: 56–62%), respectively (Table 1).

#### PCR (FQ-PCR and RT-PCR)

**Figure 13:**
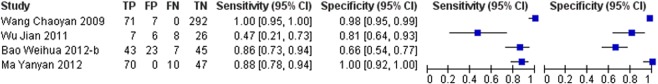
Forest plots of sensitivity and specificity for PCR. The pooled sensitivity was 90% (95% CI 55–99%; n = 4); pooled specificity of all studies was 93% (95% CI 71–99%; n = 4). TP = true positive; FP = false positive; FN = false negative; TN = true negative. The point estimates of sensitivity and specificity from each study are shown as solid circles (blue square). Error (black horizontal line) bars indicate 95% CI. Numbers indicate the studies included in the meta-analysis, as cited in the reference list.

**Figure 14:**
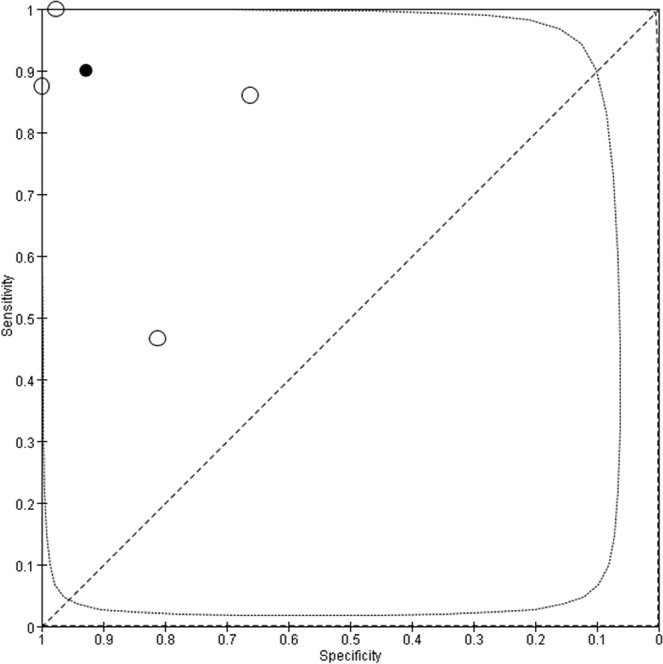
Summary receiver operating characteristic (SROC) curves for PCR. Each individual study is represented by an empty circle. The size of the circle is proportional to the sample size of the study. The solid circles correspond to the summary estimates of sensitivity and specificity and are shown with 95% confidence regions (dotted lines) and 95% prediction regions (dashed lines).

Four studies (664 participants) that reported the accuracy of PCR (FQ-PCR and RT-PCR) were analyzed. Two of these studies used products from DAAN Gene Co., China and the other two used products from PG Biotech Shenzhen, China. The sensitivities and specificities of the overall diagnostic accuracy of PCR varied widely across studies, with pooled estimates of 90% (95% CI: 55–99%) and 93% (95% CI: 71–99%) (Figs 13, 14).

For the sputum smear status of the research objects, only one study focused on smear-positive and smear-negative patients. The sensitivity of smear-positive TB was 95% (95% CI: 87–99%) (Table 1), while the sensitivity and specificity of smear-negative TB were 61% (95% CI: 36–83%) and 100% (95% CI: 92–100%) (Table 1).

### Sensitivity analyses

We assessed the results of excluding studies that were judged as high or unclear concerns for applicability in the patient selection domain, did not enroll consecutive or random patients, or were published in Chinese. The sensitivity analyses applied to these results made little difference to any of the findings (Appendix 5, Table 1).

## Discussion

In this systematic review, we estimated the diagnostic accuracy of six commercial pulmonary TB molecular diagnostic methods approved by CFDA, including Xpert MTB/RIF, LAMP, LPA, CPA, SAT-TB, and PCR. Xpert MTB/RIF possessed the highest pooled sensitivity (91%, 95% CI: 87–94%), and CPA presented the highest pooled specificity (97%, 95% CI: 95–99%), while SAT-TB had the lowest pooled sensitivity and specificity (79%, 95% CI: 66–88%; 72%, 95% CI: 48–88%). LAMP, PCR, and LPA also showed a good diagnostic performance, the pooled sensitivity and specificity of both were above or near 90% in the meta-analysis. Overall, Xpert MTB/RIF, LAMP, LPA, CPA, and PCR demonstrated high accuracy for the identification of pulmonary TB, while SAT-TB exhibited poor performance. Using RNA, which is only present in live Mycobacterium tuberculosis and degrades after the death of the bacteria^10^, as the detection target may cause poor performance of SAT-TB. In subgroup analyses, molecular diagnostics demonstrated higher sensitivity for pulmonary TB detection on smear-positive specimens.

We found that the pooled sensitivity for Xpert MTB/RIF in our review was similar to the results of previous reviews^3,4^, while the results of the pooled specificity of Xpert MTB/RIF were lower than those of previous reviews (99%, 95% CI: 98–99%^4^; 98.4%, 95% CI: 98–98.7%^3^). This may be due to the differences in study population and reference standards. Other reviews have focused on multi-ethnic populations. Additionally, in previous reviews, most studies used liquid cultures for the reference standards (n = 18, 100%^3^; n = 33, 92%^4^), but liquid cultures were only used in 35% of studies encompassed by this review. This may have reduced the specificity of the diagnostic methods^11^. The pooled sensitivity and specificity of LAMP were consistent with the findings of previous reviews^12^, even in those aimed at multi-ethnic populations. A similar condition was observed for LPA^13^, although only one study reported the accuracy of this assay. CPA and SAT-TB are both relatively new techniques for the detection of TB that have been developed by Chinese manufacturers, so no previous reviews were available with studies that used them. The sensitivity of PCR was higher than that was found in a previous review^14^ (80.8%, 95% CI: 75.8–85%), while the specificity was lower than a previous one (99%, 95% CI: 98.1–99.4%). The previous review also recruited multi-ethnic populations, which may have resulted in different results.

In subgroup analyses, molecular diagnostics demonstrated greater sensitivity for pulmonary TB detection in smear-positive specimens. The results of this study are in line with the results of all previous reviews^3,12,13,15^. This may be because smear-positive specimens have higher bacterial loads of pulmonary TB, meaning that the use of such specimens may lead to a higher sensitivity, relative to smear-negative specimens^3^.

This review provided an estimate of the diagnostic accuracy of six commercial molecular diagnostic methods for pulmonary TB. However, when physicians choose a method, they must consider the complexity of the method in addition to its accuracy. Xpert MTB/RIF is a rapid, automated test that is simple to perform and requires minimal biosafety measures^16^, and the cost per cartridge is $9.98^17^, so Xpert MTB/RIF is easy to promote. It was endorsed by the WHO in 2013 for use in the diagnosis of pulmonary TB^4^. LPA is another rapid molecular diagnostics, but it is more technically complex and expensive (it was designed for reference or regional laboratory settings, and cost $20–21 per test^17^) and takes longer to use than the Xpert MTB/RIF assay^13^. LAMP is a simple isothermal DNA amplification method that does not require an expensive thermocycler or detection system and allows visual detection of amplification. It is a cost-effective diagnostic method and the cost per test is $16^18^. These features allow it to be used at lower levels of the health system^19^. CPA is a new nucleic acid amplification technique for the detection of TB, and it can amplify target nucleic acid under isothermal conditions without requiring specialized equipment. CPA cost $4–5 per test, so it is suitable for use in resource-limited settings^20^. SAT-TB is a new type of nucleic acid detection technology, and it does not require the use of expensive specialized detection equipment. It can be performed on real-time PCR instruments, which are typically found in clinical laboratories^21^. RT-PCR and FQ-PCR have the advantage of rapidity, but they require high-quality clinical materials without any components that interfere with DNA amplification^22^. Beside, RT-PCR cost $26 per test^23^ and FQ-PCR cost $16.3 per test^24^. Thus, they are more complex and expensive than Xpert MTB/RIF.

This study had the following limitations: First, the results of the quality evaluation showed that many studies had uncertain bias risk assessments. Second, many studies published in Chinese were not standardized and lacked relevant information that could influence our outcomes, such as information on patients’ past history of TB and their treatment status. Third, the number of studies that used the same sample to report the accuracy of different molecular diagnostic methods was very small, so we cannot directly compare the methods. Fourth, many studies did not report basic information on their study subjects, such as their ages and HIV status. Fifth, our review only focused on the accuracy of molecular diagnostic methods, so we cannot produce comprehensive evaluation indexes, of factors such as cost effectiveness and manipulability. Finally, many other factors that can cause heterogeneity in a systematic review of diagnostic tests were not further analyzed in the subgroups due to the lack of the information needed for bivariate analyses, such as population (e.g., patient age), sample type, and design method. Thus, our pooled estimates must be interpreted with caution.

## Methods

This protocol has been registered with the international prospective register of systematic reviews (PROSPERO) as number CRD42018093417.

### Data sources and search strategy

We performed a systematic literature search of CNKI, Wanfang, SinoMed, and VIP for reports published in Chinese, and we searched Pubmed, Embase, and the Cochrane Library for reports published in English from January 1, 2000 to September 1, 2017. The search method is given in Appendix 1. We included studies that assessed the accuracy of molecular diagnosis of pulmonary TB and downloaded their results into EndNote X8.

Two reviewers independently screened the studies in two-steps, checking the title and abstract first, and then, checking the full text according to the following inclusion criteria: whether the study design was a typical cross-sectional, randomized controlled trials (RCTs) or cohort studies; whether the study objects were Chinese adults or children presumed (or suspected) to have pulmonary TB; whether the molecular diagnosis methods used had been endorsed by CFDA as index tests; whether the target disease was pulmonary TB; whether the reference standards were MTB culture methods (solid or liquid culture for M. tuberculosis); and whether comparisons were done between molecular diagnosis and an acceptable reference standard, with the value of the true positive, true negative, false positive, and false negative reported. Any disagreement was resolved through discussion and consensus or, if necessary, the decision of a third reviewer.

### Data extraction and quality assessment

Two researchers independently conducted data extraction and quality assessment, and any disagreements were resolved by discussion with a third researcher. The extraction agree with a Cochrane review^4^, including basic information, study design, patient characteristics, disease characteristics, reference standard, index test, and outcome measures. The full list of variables is detailed in Appendix 2.

We assessed the quality of the selected studies and the potential risk of bias using the Quality Assessment of Diagnostic Accuracy Studies (QUADAS-2). This assessment has four domains: patient selection, index test, reference standard, and flow and timing. All domains were used to judge the risk of bias as low, high, or unclear, and the first three domains were judged in relation to applicability concerns. If a given domain contained one or more key questions answered by no or unclear, then it was considered to be an area of potential bias or applicability concerns^25^. We did not carry out a formal assessment of publication bias using funnel plots or regression tests because such techniques were not helpful for the study of the accuracy of diagnostic^26^.

### Statistical analysis

We performed descriptive analyses, and the study characteristics are represented in Appendix 3. For each index test, the sensitivity and specificity were found with the corresponding 95% CI, and then, we summarized the results in forest plots and a receiver operating characteristics plot^13,27^. When an index test was assessed by four or more different studies, pooled estimates of sensitivity and specificity were calculated using a bivariate logistic regression model, with hierarchical random effects. This approach allowed us to calculate pooled estimates while minimizing potential sources of variation caused by the imprecision of the sensitivity and specificity estimates within individual studies, the correlation between sensitivity and specificity across studies, and the variation in sensitivity and specificity between studies^28^. We used a univariate random-effects model to calculate estimates of sensitivity and specificity separately or simply described the results from each individual study for index tests that were assessed in fewer than four studies. To investigate the sources of heterogeneity, we refitted the meta-analysis model within groups defined by their smear status. To explore the robustness of the results, when sufficient studies were available, we performed sensitivity analyses, focusing on studies whose patient selection domains were low-risk for concerns regarding applicability, studies whose reference standard domains were low-risk for concerns regarding applicability, and studies published in English. All of the above analyses were conducted using the statistical software Stata13.0 and Revman5.3.

## Supplementary information

Accuracy of Commercial Molecular Diagnostics for the Detection of Pulmonary Tuberculosis in China: A Systematic Review
