# Supplementary material for: Accuracy of Commercial Molecular Diagnostics for the Detection of Pulmonary Tuberculosis in China: A Systematic Review
Source: Sci Rep. 2019 Mar 14;9:4553. doi: 10.1038/s41598-019-41074-8 (PMC6418248; doi:10.1038/s41598-019-41074-8)
Supplement: Supplementary file 1 — Accuracy of Commercial Molecular Diagnostics for the Detection of Pulmonary Tuberculosis in China: A Systematic Review [file 41598_2019_41074_MOESM1_ESM.docx]

Accuracy of Commercial Molecular Diagnostics for the Detection of Pulmonary Tuberculosis in China: A Systematic Review

Siwei Deng^1^, Yixin Sun^1^, Hui Xia^2^, Zhike Liu^1^, Le Gao^1^, Jichun Yang^1^, Yanlin Zhao^2^, Fei Huang^2^, Jingnan Feng^1^, Lixia Wang^2^, Shitong Huan^3^, Siyan Zhan^1^

**Affiliations**:

^1^ Department of Epidemiology and Biostatistics, Peking University School of Public Health, Beijing, China. ^2^ National Center for Tuberculosis Control and Prevention, Chinese Center for Disease Control and Prevention, Beijing, China. ^3^ Bill and Melinda Gates Foundation, Beijing, China.

**Corresponding author:**

Siyan Zhan, Department of Epidemiology and Biostatistics, Peking University School of Public Health, 38 Xueyuan Road, Haidian District, Beijng, 100191, P.R.China.

Email address: [siyan-zhan@bjmu.edu.cn](mailto:siyan-zhan@bjmu.edu.cn).

Telephone: 86-10-82805162

**Supporting information**

Appendix 1. Search terms and strategies of all databases.

Appendix 2. Extraction form.

Appendix 3. Characteristics of included studies, grouped by index test.

Appendix 4. Methodological quality assessment for each included study.

Appendix 5. Tables and Figures.

Appendix 1. Search terms and strategies of all databases

**A. English database**

- 1. PUBMED

"Tuberculosis"[Mesh] OR tuberculosis[tw] OR tuberculous[tw] OR TB[tw] OR antitubercular[tw] OR "Mycobacterium tuberculosis"[Mesh]

AND

"molecular diagnos*"[tw] OR "molecular assay*"[tw] OR "molecular diagnostic technique*"[tw] OR “Molecular Diagnostic Techniques”[Mesh]

OR

Xpert[tw] OR GeneXpert[tw] OR cepheid[tw] OR"MTB/RIF"[tw]

OR

LPA[tw] OR LPAs[tw] OR LiPA[tw] OR "GenoType MTBDR"[tw] OR Nipro[tw] OR Hain[tw] OR MTBDRsl[tw] OR MTBDRplus[tw] OR "line probe assay"[tw] OR "line probe assays"[tw] OR MTBDR*[tw]

OR

LAMP[tw] OR Loopamp[tw] OR Eiken[tw]

OR

Genechip [tw] OR Biochip[tw] OR "CapitalBio" [tw]

OR

"MeltPro TB assay" [tw] OR "Zeesan Biotech" [tw]

OR

RealAmp [tw]

OR

EasyNAT [tw] OR CPA [tw] OR Ustar[tw]

OR

"SAT-TB" [tw] OR "TB-SAT" [tw]

OR

Genprobe [tw] OR AMTD*[tw] OR MTD[tw] OR Hologic[tw]

OR

"polymerase chain reaction" [tw] OR PCR [tw] OR PCRs[tw] OR "realtime PCR" [tw] OR "careTM TB PCR assay" [tw] OR "Polymerase Chain Reaction"[Mesh]

AND

(chinese[tw] OR China[tw] OR China[Mesh])

AND

("2000/01/01"[PDAT] : "2017/09/15"[PDAT])

- 1. EMBASE

('Tuberculosis'/exp OR tuberculosis OR OR tuberculous OR TB OR antitubercular OR 'Mycobacterium tuberculosis'/exp)

AND

('molecular diagnos*' OR 'molecular assay*' OR 'molecular diagnostic testing'

OR

Xpert OR GeneXpert OR cepheid OR'MTB/RIF'

OR

LPA OR LPAs OR LiPA OR 'GenoType MTBDR' OR Nipro OR Hain OR MTBDRsl OR MTBDRplus OR 'line probe assay' OR 'line probe assays' OR MTBDR*

OR

LAMP OR Loopamp OR Eiken

OR

Genechip OR Biochip OR “CapitalBio”

OR

'MeltPro TB assay' OR Zeesan Biotech

OR

RealAmp

OR

EasyNAT OR CPA OR Ustar

OR

'SAT-TB' OR 'TB-SAT'

OR

Genprobe OR AMTD* OR MTD OR Hologic

OR

'polymerase chain reaction' OR PCR OR PCRs OR 'realtime PCR' OR 'careTM TB PCR assay')

AND

(chinese OR china OR 'china'/exp)

AND

[embase]/lim AND [2000-2017]/py

- 1. the Cochrane Library

[mh Tuberculosis] OR tuberculosis OR tuberculous OR TB OR antitubercular OR [mh "Mycobacterium tuberculosis"]

AND

"molecular diagnos*" OR "molecular assay*" OR "molecular diagnostic technique*" OR [mh “Molecular Diagnostic Techniques”]

OR

Xpert OR GeneXpert OR cepheid OR"MTB/RIF"

OR

LPA OR LPAs OR LiPA OR "GenoType MTBDR" OR Nipro OR Hain OR MTBDRsl OR MTBDRplus OR "line probe assay" OR "line probe assays" OR "molecular diagnostic technique" OR MTBDR*

OR

LAMP OR Loopamp OR Eiken

OR

Genechip OR Biochip OR “CapitalBio”

OR

"MeltPro TB assay" OR Zeesan Biotech

OR

RealAmp

OR

EasyNAT OR CPA OR Ustar

OR

"SAT-TB" OR "TB-SAT"

OR

Genprobe OR AMTD* OR MTD OR Hologic

OR

"polymerase chain reaction" OR PCR OR PCRs OR "realtime PCR" OR "careTM TB PCR assay"

AND

(chinese OR China OR [mh China])

AND

("2000"[PDAT] : "2017"[PDAT])

**B. Chinese database**

1. VIP

(M=活动性肺结核 + M=耐药肺结核+ M=肺结核)*(M=分子检测 + M=分子诊断 + M=Xpert + M=MTB/RIF + M=GeneXpert + M=实时荧光环 + M=GenoType MTBDR + M=MTBDRsl + M=MTBDRplus + M=线性探针技术 + M=LAMP + M=Loopamp + M=蓝谱 + M=日本荣研 + M=LPA + M=Genechip + M=Biochip + M=基因芯片 + M=DNA微阵列芯片 + M=博奥 + M=MeltPro TB assay + M=致善生物 + M=RealAmp + M=恒温扩增 + M=EasyNAT + M=CPA + M=恒温扩增-试纸条 + M=优思达 + M=SAT + M=TB-SAT + M=RNA恒温扩增 + M=环介导恒温扩增 + M=Geneprobe AMTD + M=MTD + M=TMA杂交 + M=豪洛捷 + M=PCR + M=荧光 + M=荧光PCR + M=荧光探针 + M=荧光定量PCR + M=PCR反向点杂交 + M=荧光PCR熔解曲线 + M=熔解曲线 + M=PCRSanger测序 + M=PCR测序 + M=实时荧光PCR + M=PCR线性杂交酶显色 + M=聚合酶链式反应 + M=之江 + M=克隆生物 + M=匹基 + M=泰普 + M=达安基因 + M=安普利 + M=艾康 + M=复星长征 + M=华大吉比爱 + M=圣湘 + M=仁度 + M=百泰基因 + M=普瑞康 + M=凯杰 + M=迪澳 + M=华峰 + M=东北制药集团辽宁生物医药 + M=万泰 + M=海力特 + M=永安 + M=亚能 + M=博奥晶芯)

和 时间限定：（2000/01/01-2017/09/15）

1. SinoMed

(1)("活动性肺结核" or "耐药肺结核" or "肺结核") and ("分子检测" or "分子诊断" or "Xpert" or "MTB/RIF" or "GeneXpert" or "实时荧光环" or "GenoType MTBDR" or "MTBDRsl" or "MTBDRplus" or "线性探针技术" or "LAMP" or "Loopamp" or "蓝谱" or "日本荣研" or "LPA" or "Genechip" or "Biochip" or "基因芯片" or "DNA微阵列芯片" or "博奥" or "MeltPro TB assay" or "致善生物" or "RealAmp" or "恒温扩增")

(2)("活动性肺结核" or "耐药肺结核" or "肺结核") and ("EasyNAT" or "CPA" or "恒温扩增-试纸条" or "优思达" or "SAT" or "TB-SAT" or "RNA恒温扩增" or "环介导恒温扩增" or "Geneprobe AMTD" or "MTD" or "TMA杂交" or "豪洛捷" or "PCR" or "荧光" or "荧光PCR" or "荧光探针" or "荧光定量PCR" or "PCR-反向点杂交" or "荧光PCR熔解曲线" or "熔解曲线" or "PCR-Sanger测序" or "PCR-测序" or "实时荧光PCR")

(3) ("活动性肺结核" or "耐药肺结核" or "肺结核") and ("PCR-线性杂交酶显色" or "聚合酶链式反应" or "之江" or "克隆生物" or "匹基" or "泰普" or "达安基因" or "安普利" or "艾康" or "复星长征" or "华大吉比爱" or "圣湘" or "仁度" or "百泰基因" or "普瑞康" or "凯杰" or "迪澳" or "华峰" or "东北制药集团辽宁生物医药" )

(4) ("活动性肺结核" or "耐药肺结核" or "肺结核") and ("万泰" or "海力特" or "永安" or "亚能" or "博奥晶芯")

和时间限定：（2000-2017）

1. WanFang

(1) 主题:(活动性肺结核 + 耐药肺结核 + 肺结核) * 主题:(分子检测 + 分子诊断 + Xpert + MTB + RIF + GeneXpert + 实时荧光环 + "GenoType MTBDR" + MTBDRsl + MTBDRplus + 线性探针技术 + LAMP + Loopamp + 蓝谱 + 日本荣研 + LPA + Genechip + Biochip + 基因芯片 + "DNA微阵列芯片")

*Date:2000-2017

(2) 主题:(活动性肺结核 + 耐药肺结核 + 肺结核) * 主题:（PCR + 荧光 + "荧光PCR" + 荧光探针 + "荧光定量PCR" + PCR-反向点杂交 + 荧光PCR熔解曲线 + 熔解曲线 + "PCR-Sanger测序" + PCR-测序 + "实时荧光PCR" + PCR-线性杂交酶显色)

*Date :2000-2017

(3) 主题:(活动性肺结核 + 耐药肺结核 + 肺结核) * 主题:（聚合酶链式反应+博奥 + "MeltPro TB assay" + 致善生物 + "RealAmp" + 恒温扩增 + EasyNAT + CPA + 恒温扩增-试纸条 + 优思达+ SAT + TB-SAT + "RNA恒温扩增" + 环介导恒温扩增 + "Geneprobe AMTD" + MTD + "TMA杂交" + 豪洛捷）

*Date :2000-2017

(4) 主题:(活动性肺结核 + 耐药肺结核 + 肺结核) * 主题:("之江"+ "克隆生物" + "匹基" + "泰普" + "达安基因" + "艾康" + "复兴长征" + "华大吉比爱" + "圣湘" + "仁度" + "百泰基因" + "普瑞康" + "凯杰" + "迪澳" + "华峰" + "万泰" + "海力特" + "永安" + "亚能" + "东北制药集团辽宁生物制药" + "博奥晶芯")

*Date :2000-2017

1. CNKI

(1)SU=("活动性肺结核"+"耐药肺结核"+"肺结核")*("分子检测"+"Xpert"+"MTB"+"RIF"+"GeneXpert"+"实时荧光环"+"GenoType MTBDR"+"MTBDRsl"+"MTBDRplus"+"线性探针技术"+"LAMP"+"Loopamp"+"蓝谱"+"LPA"+"Genechip"+"Biochip"+"基因芯片"+"DNA微阵列芯片"+"MeltPro TB assay"+"RealAmp"+"恒温扩增"+"EasyNAT"+"CPA"+"恒温扩增-试纸条"+"SAT"+"TB-SAT"+"RNA恒温扩增"+"环介导恒温扩增"+"Geneprobe AMTD"+"MTD"+"TMA杂交"+"PCR"+"荧光"+"荧光PCR"+"荧光探针"+"荧光定量PCR"+"PCR-反向点杂交"+"荧光PCR熔解曲线"+"熔解曲线"+"PCR-Sanger测序"+"PCR-测序"+"实时荧光PCR"+"PCR-线性杂交酶显色"))

(2)SU=("活动性肺结核"+"耐药肺结核"+"肺结核")*("分子诊断"+"日本荣研"+"博奥"+"致善生物"+"优思达"+"豪洛捷"+"聚合酶链式反应"+"之江"+"克隆生物"+"匹基"+"泰普"+"达安基因"+"安普利"+"艾康"+"复星长征"+"华大吉比爱"+"圣湘"+"仁度"+"百泰基因"+"普瑞康"+"凯杰"+"迪澳"+"华峰"+"东北制药集团辽宁生物医药"+"万泰"+"海力特"+"永安"+"亚能"+"博奥晶芯")

并且 发表时间 between (2000-01-01,2017-09-15)

Appendix 2. Extraction form

| 1. **Identifier** |
| --- |
| Your name {A1} __________ |
| Endnote Language {A2} ____________________ |
| Endnote ID {A3} _______________ |
| 1. **Basic Information** |
| 1. Whether it meets the inclusion criteria {B1} # ①yes ②no ③ uncertain   If "no", then the exclusion reason(21,22,23,24,25,26,27,28,29,30,31,32) {B2} ##  If "uncertain", then fill in the author's name {B3} _________ and email {B4} ____________ |
| 1. First author {B5} ______________________________ |
| 1. Language {B6} # ①Chinese ②English |
| 1. Publication year {B7} #### |
| 1. Study type {B8} # ①manufacturer ②independent validation study |
| 1. **Design** |
| 1. Study design {C1} # ①Cross-sectional ②case-control ③cohort studies   ④ randomized controlled trials ⑤Unclear/not reported |
| 1. Study data collection {C2} # ①Prospective ②Retrospective ③Unclear/not reported |
| 1. Participant selection {C3} # ①Convenience ②Consecutive ③Random   ④Other Unclear/not reported |
| 1. **Patient Characteristics** |
| 1. Number of females {D1} #######/ Number of patients {D2} ######## |
| 1. Type of patients {D3}____________________________________________ |
| 1. Population {D4} # ①adult ②children(younger than 15) ③adult and children   ④unclear/not reported |
| 1. Age: Mean (standard deviation) {D5}###.##({D6}###.##)   Median (IQR) {D7}###.##({D8}###.##)  Range {D9} ______________________________ |
| 1. Concomitant disease   1. HIV {D10} # ①yes ②no ③Unclear/not reported  if yes, the percentage of participants with HIV {D11} ##.##%  2. Diabetes mellitus {D12} # ①yes ②no ③Unclear/not reported  if yes, the percentage of participants with Diabetes mellitus {D13} ##.##%  3. Pneumoconiosis {D14} # ①yes ②no ③Unclear/not reported  if yes, the percentage of participants with pneumoconiosis {D15} ##.##%  4. Lung cancer {D16} # ①yes ②no ③Unclear/not reported  if yes, the percentage of participants with lung cancer {D17} ##.##%  5. other {D18}________________________________________  if yes, the percentage of participants with other {D19} ##.##% |
| 1. Past history of TB? {D20} # ①Yes ②No ③Unclear/not reported;   if yes, percentage {D21} ##.##% |
| 1. Percent of patients on TB treatment(for > one week){D22} # ①reported ②Unclear/not reported;   if reported, percentage {D23} ##.##% |
| 1. Population region (province) {D24}________________________________________ |
| 1. **Disease Characteristics** |
| 1. Level of laboratory running the index test? {E1} # ①County ②City ③Province ④other {E2} ____ |
| 1. Disease {E3} # ①Simple active tuberculosis ②drug-resistant tuberculosis ③other {E4}________ |
| 1. **Simple active tuberculosis** |
| 1. Sample size {F1} ######## |
| 1. Specimen used(可多选) {F10} #   ①expectorated sputum  ②induced sputum  ③gastric aspirate in children  ④Unknown/Not reported  ⑤others {F11} ______________________ |
| 1. Condition of the specimen {F12} ####   ①fresh ②frozen ③Unknown/Not reported  If frozen, specify duration {F13} ______________ |
| - **Reference standard** |
| 1. Reference standard   For TB：{F2} #  ①Solid media ②liquid media ③Unknown/Not reported  If solid media, please choose which one {F2} #   1. solid media (L?wenstein-Jensen) **Löwenstein-Jensen**   ②solid media (Middlebrook 7H10)  ③solid media (Middlebrook 7H11)  ④solid media (Ogawa media)  ⑤Others {F3}__________________________________  If liquid media, please choose which one {F4} ##  ⑥commercial liquid culture system (BACTEC? 460TB System) **BACTEC™ 460TB System**  ⑦commercial liquid culture system (BACTEC?MGIT?960 Mycobacterial Detection System, BD, USA)  **BACTEC™MGIT™960 Mycobacterial Detection System, BD, USA**  ⑧commercial liquid culture system (BacT/ALERT?System, bioMérieux,France）  **BacT/ALERT® System, bioMérieux,France**  ⑨commercial liquid culture system (VersaTREK? Mycobacteria Detection & Susceptibility, Thermo Fisher Scientific, USA).  **VersaTREK® Mycobacteria Detection & Susceptibility, Thermo Fisher Scientific, USA**  ⑩Others {F5} __________________________________  speciate mycobacteria isolated in culture?  {F7} # ①yes ②no ③Unknow/Not reported |
| - **Index test** |
| 1. Index test: {F8} ____________________ |
| 1. Index test manufacturer {F9} ___________________________________ |
| - **Outcome** |
| 1. Outcome measures   True positive {TP4} #######  False positive {FP4} #######  False negative {FN4} #######  True negative {TN4} #######  Number of indeterminate or without results: |
| - Smear-positives {Z1} # ①yes ②no   True positive {TP2} #######  False positive {FP2} #######  False negative {FN2} #######  True negative {TN2} #######  Number of indeterminate or without results: |
| - Smear-negatives {Z2} # ①yes ②no   True positive {TP3} #######  False positive {FP3} #######  False negative {FN3} #######  True negative {TN3} #######  Number of indeterminate or without results: |

Appendix 3. Characteristics of included studies, grouped by index test

Table S1. Basic characteristics of included studies for active TB

| **Study ID** | **Language** | **Study design** | **Adult or children** | **HIV or not** | **Level of lab** | **Province** | **Sample size** | **Past history of TB** | **Reference standard** | **Index test** | **Manufacture** |
| --- | --- | --- | --- | --- | --- | --- | --- | --- | --- | --- | --- |
| Bao Weihua 2012 | Chinese | Cross-sectional | NA | NA | City | Guangxi | 118 | U | Solid media | LAMP | Guangzhou Huafeng Biotech |
| Bao Weihua 2012 | Chinese | Cross-sectional | NA | NA | City | Guangxi | 118 | U | Solid media | PCR | DAAN Gene Co |
| Ceng Songfang 2016 | Chinese | Cross-sectional | NA | NA | County | Zhejiang | 200 | U | Solid media | Xpert | Cepheid, USA |
| Chen Wei 2017 | Chinese | Cross-sectional | both | NA | County | Guizhou | 1590 | U | Solid media | Xpert | Cepheid, USA |
| Chen Xiuqiong 2012 | Chinese | Cohort studies | NA | NA | City | Guangdong | 54 | U | NA | LAMP | Guangzhou Di'ao Biotech |
| Guo Chixing 2016 | Chinese | Cross-sectional | adult | NA | City | Guangdong | 215 | U | NA | Xpert | Cepheid, USA |
| Huang Jianbin 2017 | Chinese | Cross-sectional | NA | NA | City | Hebei | 92 | U | Solid media | Xpert | Cepheid, USA |
| Jiang Xiaoying 2017 | Chinese | Cross-sectional | NA | NA | City | Twelve provinces(Beijing/Tianjin/Hebei/Shaanxi/Liaoning,etc) | 1020 | no | Solid media | CPA | EasyNAT® TB |
| Li Hui 2015 | Chinese | Cross-sectional | both | NA | County | Henan | 1356 | U | Solid media | CPA | EasyNAT® TB |
| Li Junlian 2017 | Chinese | Cohort studies | adult | NA | Province | Xinjiang | 2381 | no | Solid media | SAT-TB | Shanghai Rendu Biotechnology |
| Li Qiang 2016 | Chinese | Cohort studies | NA | NA | Province | Anhui/Shaanxi | 1973 | U | Liquid media | LPA | Hain Lifescience GmbH, Nehren, Germany |
| Liu Aimei 2017 | Chinese | Cross-sectional | both | NA | City | Guangxi | 851 | U | Solid media | Xpert | Cepheid, USA |
| Liu Jianxia 2016 | Chinese | Cohort studies | NA | NA | Province | Tianjin | 185 | U | Solid media | Xpert | Cepheid, USA |
| Liu Tao 2017 | Chinese | Cohort studies | both | NA | County | Shanghai | 430 | no | Liquid media | Xpert | Cepheid, USA |
| Liu Yaqin 2015 | Chinese | Cross-sectional | adult | NA | City | Shandong | 413 | U | Solid media | Xpert | Cepheid, USA |
| Ma Yanyan 2012 | Chinese | Cross-sectional | NA | NA | Province | Henan | 129 | U | Solid media | PCR | PG Biotech Shenzhen |
| Mao Xiujun 2016 | Chinese | Cohort studies | adult | NA | City | Hebei | 206 | U | Solid media | CPA | EasyNAT® TB |
| Ou Xichao 2016 | Chinese | Cohort studies | NA | NA | County | Henan | 1323 | no | Solid media | LAMP | Eiken Chemical Co, Japan |
| Rendong Fang, 2009 | English | Cohort studies | NA | NA | Province | Shanghai | 180 | U | Liquid media | CPA | EasyNAT® TB |
| Sha Wei 2012 | Chinese | Cohort studies | both | NA | Province | Shanghai | 172 | U | Solid media | SAT-TB | Shanghai Rendu Biotechnology |
| Sheng Fen Wang 2016 | English | Cross-sectional | NA | NA | County | Beijing | 270 | U | Solid media | Xpert | Cepheid, USA |
| Tingyu Tang 2017 | English | Cross-sectional | NA | NA | Province | Zhejiang | 240 | U | Liquid media | Xpert | Cepheid, USA |
| Wang Chaoyan 2009 | Chinese | Cohort studies | NA | NA | City | Hunan | 370 | U | Liquid media | PCR | PG Biotech Shenzhen |
| Wei Xuantong 2016 | Chinese | Cohort studies | NA | NA | Province | Guangxi | 353 | U | Solid media | Xpert | Cepheid, USA |
| Wu Jian 2011 | Chinese | Cross-sectional | NA | NA | Province | Guangdong | 47 | U | Liquid media | PCR | DAAN Gene Co |
| Xia Qiang 2014 | Chinese | Cohort studies | NA | NA | Province | Zhejiang | 386 | U | Liquid media | SAT-TB | Shanghai Rendu Biotechnology |
| Xichao Ou 2014 | English | Cross-sectional | NA | NA | Province | Henan | 1329 | U | Solid media | LAMP | NA |
| Xichao Ou 2014 | English | Cross-sectional | NA | NA | Province | Beijing | 2200 | U | Solid media | CPA | NA |
| Xichao Ou 2016 | English | Cross-sectional | both | NA | City | Shandong、Shanghai、Guangdong | 1583 | U | Liquid media | LAMP | NA |
| Ye Peng 2017 | Chinese | Cohort studies | adult | NA | City | Henan | 215 | U | Liquid media | Xpert | Cepheid, USA |
| Yu Pang 2014 | English | Cross-sectional | children | NA | County | Beijing | 211 | no | Liquid media | Xpert | Cepheid, USA |
| Yu Xia 2013 | Chinese | Cohort studies | adult | NA | Province | Beijing | 246 | U | Solid media | LAMP | Eiken Chemical Co, Japan |
| Zhang Canqiang 2016 | Chinese | Cohort studies | adult | NA | County | Guangdong | 147 | U | Solid media | Xpert | Cepheid, USA |
| Zhang Guoqin 2017 | Chinese | Cohort studies | both | NA | Province | Tianjin | 504 | U | Liquid media | Xpert | Cepheid, USA |
| Zhang Qing 2016 | Chinese | Cohort studies | NA | NA | City | Tianjin | 99 | U | Solid media | Xpert | Cepheid, USA |
| Zhang Yan 2016 | Chinese | Cohort studies | adult | NA | City | Zhejiang | 200 | U | Solid media | Xpert | Cepheid, USA |
| Zhengwei Liu 2017 | English | Cohort studies | NA | NA | County | Zhejiang | 3151 | U | Solid media | Xpert | Cepheid, USA |
| Zhong Liyun 2017 | Chinese | Cohort studies | NA | NA | County | Guangdong | 110 | U | Solid media | Xpert | Cepheid, USA |
| Zhu Yankun 2016 | Chinese | Cohort studies | NA | NA | County | Henan | 916 | no | NA | Xpert | Cepheid, USA |
| Zhu Yankun 2016 | Chinese | Cohort studies | NA | NA | County | Henan | 673 | no | NA | CPA | EasyNAT® TB |
| Zhu Yankun 2016 | Chinese | Cohort studies | NA | NA | County | Henan | 916 | no | NA | CPA | EasyNAT® TB |

**Notes：** U-unclear；Y-yes；LJ-Löwenstein-Jensen；BM-960--BACTEC™MGIT™960；Xpert -Xpert MTB/RIF

Appendix 4. Methodological quality assessment for each included study

Table S2. Methodological quality assessment for each included study.

| **Study** | **RISK OF BIAS** | | | | **APPLICABILITY CONCERNS** | | |
| --- | --- | --- | --- | --- | --- | --- | --- |
|  | **PATIENT SELECTION** | **INDEX TEST** | **REFERENCE STANDARD** | **FLOW AND TIMING** | **PATIENT SELECTION** | **INDEX TEST** | **REFERENCE STANDARD** |
| Bao Weihua(2012) | ? | ☺ | ? | ? | ☺ | ☺ | ? |
| Bao Weihua(2012) | ? | ☺ | ? | ? | ☺ | ☺ | ? |
| Ceng Songfang(2016) | ? | ☺ | ? | ? | ☺ | ☺ | ☺ |
| Chen Wei(2017) | ? | ☺ | ? | ☺ | ☺ | ☺ | ☺ |
| Chen Xiuqiong(2012) | ? | ☺ | ? | ? | ☺ | ☺ | ☺ |
| Guo Chixing(2016) | ? | ☺ | ? | ? | ☺ | ☺ | ☺ |
| Huang Jianbin(2017) | ? | ☺ | ? | ? | ☺ | ☺ | ☺ |
| Jiang Xiaoying(2017) | ☹ | ☺ | ? | ☺ | ☹ | ☺ | ☺ |
| Li Hui(2015) | ☹ | ☺ | ? | ? | ☹ | ☺ | ☺ |
| Li Junlian(2017) | ☹ | ☺ | ? | ☺ | ☹ | ☺ | ? |
| Li Qiang(2016) | ☹ | ☺ | ? | ☺ | ☹ | ☺ | ☺ |
| Liu Aimei(2017) | ☺ | ☺ | ? | ? | ☺ | ☺ | ☺ |
| Liu Jianxia(2016) | ? | ☺ | ? | ? | ☺ | ☺ | ☺ |
| Liu Tao(2017) | ? | ☺ | ? | ? | ☺ | ☺ | ☺ |
| Liu Yaqin(2015) | ? | ☺ | ? | ? | ☺ | ☺ | ☺ |
| Ma Yanyan(2012) | ? | ☺ | ? | ? | ☺ | ☺ | ☺ |
| Mao Xiujun(2016) | ☺ | ☺ | ? | ? | ☺ | ☺ | ☺ |
| Ou Xichao(2016) | ? | ☺ | ? | ? | ☺ | ☺ | ☺ |
| Rendong Fang,(2009) | ? | ☺ | ? | ? | ☺ | ☺ | ☺ |
| Sha Wei(2012) | ? | ☺ | ? | ? | ☺ | ☺ | ☺ |
| Sheng Fen Wang(2016) | ? | ☺ | ? | ? | ☺ | ☺ | ☺ |
| Tingyu Tang(2017) | ☺ | ☺ | ? | ? | ☺ | ☺ | ☺ |
| Wang Chaoyan(2009) | ? | ☺ | ? | ? | ☺ | ☺ | ? |
| Wei Xuantong(2016) | ? | ☺ | ? | ? | ☺ | ☺ | ☺ |
| Wu Jian(2011) | ☹ | ☺ | ? | ☺ | ☹ | ☺ | ☺ |
| Xia Qiang(2014) | ☹ | ☺ | ? | ? | ☹ | ☺ | ☺ |
| Xichao Ou(2016) | ☺ | ☺ | ☺ | ? | ☺ | ☺ | ☺ |
| Xichao Ou(2014) | ☺ | ☺ | ? | ? | ☺ | ☺ | ☺ |
| Xichao Ou(2014) | ☺ | ☺ | ? | ? | ☺ | ☺ | ☺ |
| Ye Peng(2017) | ? | ☺ | ? | ☺ | ☺ | ☺ | ☺ |
| Yu Pang(2014) | ☺ | ☺ | ? | ? | ☺ | ☺ | ☺ |
| Yu Xia(2013) | ? | ☺ | ? | ☹ | ☺ | ☺ | ☺ |
| Zhang Canqiang(2016) | ? | ☺ | ? | ? | ☺ | ☺ | ? |
| Zhang Guoqin(2017) | ? | ☺ | ? | ☺ | ☺ | ☺ | ☺ |
| Zhang Qing(2016) | ? | ☺ | ? | ? | ☺ | ☺ | ☺ |
| Zhang Yan(2016) | ☹ | ☺ | ? | ? | ☹ | ☺ | ? |
| Zhengwei Liu(2017) | ☺ | ☺ | ? | ? | ☺ | ☺ | ☺ |
| Zhong Liyun(2017) | ? | ☺ | ? | ? | ☺ | ☺ | ☺ |
| Zhu Yankun(2016) | ☺ | ☺ | ? | ? | ☺ | ☺ | ? |
| Zhu Yankun(2016) | ☺ | ☺ | ? | ? | ☺ | ☺ | ? |
| Zhu Yankun(2016) | ☺ | ☺ | ? | ? | ☺ | ☺ | ? |

☺low risk ☹high risk ? unclear

Table S3. Signalling questions results of QUADAS-2 for active TB

| **Study** | **Risk of bias** | | | | | | | | | | |  | **Applicability** | | |
| --- | --- | --- | --- | --- | --- | --- | --- | --- | --- | --- | --- | --- | --- | --- | --- |
|  | **PATIENT SELECTION** | | | **INDEX TEST** | | **REFERENCE STANDARD** | | **FLOW AND TIMING** | | | |  | **PATIENT SELECTION** | **INDEX TEST** | **REFERENCE STANDARD** |
|  | **Q1** | **Q2** | **Q3** | **Q1** | **Q2** | **Q1** | **Q2** | **Q1** | **Q2** | **Q3** | **Q4** |  |  |  |  |
| Bao Weihua(2012) | U | Y | Y | Y | Y | U | U | U | Y | Y | Y |  | Low | Low | U |
| Bao Weihua(2012) | U | Y | Y | Y | Y | U | U | U | Y | Y | Y |  | Low | Low | U |
| Ceng Songfang(2016) | U | Y | Y | Y | Y | Y | U | U | Y | Y | Y |  | Low | Low | Low |
| Chen Wei(2017) | U | Y | Y | Y | Y | Y | U | Y | Y | Y | Y |  | Low | Low | Low |
| Chen Xiuqiong(2012) | U | Y | Y | Y | Y | Y | U | U | Y | Y | Y |  | Low | Low | Low |
| Guo Chixing(2016) | U | Y | Y | Y | Y | Y | U | U | Y | Y | Y |  | Low | Low | Low |
| Huang Jianbin(2017) | U | Y | Y | Y | Y | Y | U | U | Y | Y | Y |  | Low | Low | Low |
| Jiang Xiaoying(2017) | U | Y | N | Y | Y | Y | U | Y | Y | Y | Y |  | High | Low | Low |
| Li Hui(2015) | Y | Y | N | Y | Y | Y | U | U | Y | Y | Y |  | High | Low | Low |
| Li Junlian(2017) | Y | Y | N | Y | Y | U | U | Y | Y | Y | Y |  | High | Low | U |
| Li Qiang(2016) | Y | Y | N | Y | Y | Y | U | Y | Y | Y | Y |  | High | Low | Low |
| Liu Aimei(2017) | Y | Y | Y | Y | Y | Y | U | U | Y | Y | Y |  | Low | Low | Low |
| Liu Jianxia(2016) | U | Y | Y | Y | Y | Y | U | U | Y | Y | Y |  | Low | Low | Low |
| Liu Tao(2017) | U | Y | Y | Y | Y | Y | U | U | Y | Y | Y |  | Low | Low | Low |
| Liu Yaqin(2015) | U | Y | Y | Y | Y | Y | U | U | Y | Y | Y |  | Low | Low | Low |
| Ma Yanyan(2012) | U | Y | Y | Y | Y | Y | U | U | Y | Y | Y |  | Low | Low | Low |
| Mao Xiujun(2016) | Y | Y | Y | Y | Y | Y | U | U | Y | Y | Y |  | Low | Low | Low |
| Ou Xichao(2016) | U | Y | Y | Y | Y | Y | U | U | Y | Y | Y |  | Low | Low | Low |
| Rendong Fang,(2009) | U | Y | Y | Y | Y | Y | U | U | Y | Y | Y |  | Low | Low | Low |
| Sha Wei(2012) | U | Y | Y | Y | Y | Y | U | U | Y | Y | Y |  | Low | Low | Low |
| Sheng Fen Wang(2016) | U | Y | Y | Y | Y | Y | U | U | Y | Y | Y |  | Low | Low | Low |
| Tingyu Tang(2017) | Y | Y | Y | Y | Y | Y | U | U | Y | Y | Y |  | Low | Low | Low |
| Wang Chaoyan(2009) | U | Y | Y | Y | Y | U | U | U | Y | Y | Y |  | Low | Low | U |
| Wei Xuantong(2016) | U | Y | Y | Y | Y | Y | U | U | Y | Y | Y |  | Low | Low | Low |
| Wu Jian(2011) | U | Y | N | Y | Y | Y | U | Y | Y | Y | Y |  | High | Low | Low |
| Xia Qiang(2014) | U | Y | N | Y | Y | Y | U | U | Y | Y | Y |  | High | Low | Low |
| Xichao Ou(2016) | Y | Y | Y | Y | Y | Y | Y | U | Y | Y | Y |  | Low | Low | Low |
| Xichao Ou(2014) | Y | Y | Y | Y | Y | Y | U | U | Y | Y | Y |  | Low | Low | Low |
| Xichao Ou(2014) | Y | Y | Y | Y | Y | Y | U | U | Y | Y | Y |  | Low | Low | Low |
| Ye Peng(2017) | U | Y | Y | Y | Y | Y | U | Y | Y | Y | Y |  | Low | Low | Low |
| Yu Pang(2014) | Y | Y | Y | Y | Y | Y | U | U | Y | Y | Y |  | Low | Low | Low |
| Yu Xia(2013) | U | Y | Y | Y | Y | Y | U | U | Y | Y | N |  | Low | Low | Low |
| Zhang Canqiang(2016) | U | Y | Y | Y | Y | U | U | U | Y | Y | Y |  | Low | Low | U |
| Zhang Guoqin(2017) | U | Y | Y | Y | Y | Y | U | Y | Y | Y | Y |  | Low | Low | Low |
| Zhang Qing(2016) | U | Y | Y | Y | Y | Y | U | U | Y | Y | Y |  | Low | Low | Low |
| Zhang Yan(2016) | U | Y | N | Y | Y | U | U | U | Y | Y | Y |  | High | Low | U |
| Zhengwei Liu(2017) | Y | Y | Y | Y | Y | Y | U | U | Y | Y | Y |  | Low | Low | Low |
| Zhong Liyun(2017) | U | Y | Y | Y | Y | Y | U | U | Y | Y | Y |  | Low | Low | Low |
| Zhu Yankun(2016) | Y | Y | Y | Y | Y | U | U | U | Y | Y | Y |  | Low | Low | U |
| Zhu Yankun(2016) | Y | Y | Y | Y | Y | U | U | U | Y | Y | Y |  | Low | Low | U |
| Zhu Yankun(2016) | Y | Y | Y | Y | Y | U | U | U | Y | Y | Y |  | Low | Low | U |

**Notes: Y-yes; N-no; U-unclear**

Appendix 5. Sensitivity analyses

Table S4. Results of sensitivity analyses.

| Molacular diagnostics | No. of studies  (After excluding) | Pooled Sensitivity  [95% CI] | Pooled Specificity  [95% CI] | No. of studies  (overall) | Pooled Sensitivity  [95% CI] | Pooled Specificity  [95% CI] |
| --- | --- | --- | --- | --- | --- | --- |
| ***Excluding studies that were judged as high or unclear concerns of applicability in the patient selection domain.*** | | | | | | |
| Xpert MTB/RIF | 19 | 0.91[0.87,0.94] | 0.91[0.88,0.93] | 20 | 0.91[0.87,0.94] | 0.92[0.89,0.94] |
| CPA | 5 | 0.87[0.83,0.91] | 0.97[0.96,0.98] | 7 | 0.87[0.84,0.89] | 0.97[0.95,0.99] |
| ***Excluding studies that did not enroll a consecutive or random patients.*** | | | | | | |
| Xpert MTB/RIF | 5 | 0.88[0.79,0.95] | 0.91[0.83,0.95] | 20 | 0.91[0.87,0.94] | 0.92[0.89,0.94] |
| ***Excluding studies that published in Chinese.*** | | | | | | |
| Xpert MTB/RIF | 4 | 0.88[0.71,0.96] | 0.88[0.78,0.94] | 20 | 0.91[0.87,0.94] | 0.92[0.89,0.94] |
| CPA | 4 | 0.87[0.82,0.90] | 0.98[0.96,0.99] | 7 | 0.87[0.84,0.89] | 0.97[0.95,0.99] |
